# CD10^+^ Cells and IgM in Pathogen Response in Lumpfish (*Cyclopterus lumpus*) Eye Tissues

**DOI:** 10.3389/fimmu.2020.576897

**Published:** 2020-11-20

**Authors:** Robert L. Gendron, Hélène Paradis, Raahyma Ahmad, Kenneth Kao, Danny Boyce, William V. Good, Surendra Kumar, Ignacio Vasquez, Trung Cao, Ahmed Hossain, Setu Chakraborty, Katherinne Valderrama, Javier Santander

**Affiliations:** ^1^Division of Biomedical Sciences, Faculty of Medicine, Memorial University, St. John’s, NL, Canada; ^2^Marine Microbial Pathogenesis and Vaccinology Lab, Department of Ocean Sciences, Memorial University, St. John’s, NL, Canada; ^3^Smith Kettlewell Eye Research Institute, San Francisco, CA, United States

**Keywords:** lumpfish (*Cyclopterus lumpus* L.), eye, immune response, infection, cluster of differentiation 10, immunoglobulin M

## Abstract

Lumpfish (*Cyclopterus lumpus*), a North Atlantic “cleaner“ fish, is utilized to biocontrol salmon louse (*Lepeophtheirus salmonis*) in Atlantic salmon (*Salmo salar*) farms. Lumpfish require excellent vision to scan for and eat louse on salmon skin. The lumpfish eye immune response to infectious diseases has not been explored. We examined the ocular response to a natural parasite infection in wild lumpfish and to an experimental bacterial infection in cultured lumpfish. Cysts associated with natural myxozoan infection in the ocular scleral cartilage of wild adult lumpfish harbored cells expressing cluster of differentiation 10 (CD10) and immunoglobulin M (IgM). Experimental *Vibrio anguillarum* infection, which led to exophthalmos and disorganization of the retinal tissues was associated with disruption of normal CD10 expression, CD10^+^ cellular infiltration and IgM expression. We further describe the lumpfish CD10 orthologue and characterize the lumpfish scleral skeleton in the context of myxozoan scleral cysts. We propose that lumpfish develop an intraocular response to pathogens, exemplified herein by myxozoan and *V. anguillarum* infection involving novel CD10^+^ cells and IgM^+^ cells to contain and mitigate damage to eye structures. This work is the first demonstration of CD10 and IgM expressing cells in a novel ocular immune system component in response to disease in a teleost.

## Introduction

The aquaculture and utilization of lumpfish as cleaner fish to control sea-lice infestation in the Atlantic salmon (*Salmo salar*) industry has become commercially relevant in the last 10 years (1–5), making the study and protection of this species increasingly important. Sea-lice are copepod ectoparasites that immune compromise the fish host, increasing susceptibility to infections (6), resulting in significant losses and high treatment costs (7–9).

The literature on disease causing infection in lumpfish has been growing over the past decade (10–12), indicating the significant environmental threats that lumpfish encounter in the wild. Lumpfish recently have been designated as near-threatened on a global basis and as threatened in the Northwest Atlantic in Canada (13); Committee on the Status of Endangered Wildlife in Canada [COSEWIC], Government of Canada). New knowledge of inherent features that participate in response to exogenous threats to the eyes of lumpfish is required to better understand their survival in the wild and/or use as a cleaner fish.

Pelagic trawl records and videos suggest that, while lumpfish reside mainly in the upper 60 m of the ocean, they can also be frequently found up to 498 m with daily vertical migrations greater than 100 m (14–18). The range in habitat across depths containing large variations in light levels, temperature and hydrostatic pressure might have forced evolution of novel morphological changes, ostensibly offering survival advantages to this species.

Lumpfish have a scan-and-pick sea-lice feeding behavior that requires sophisticated anatomical and functional features in their visual system. We have described novel accessory retinal tissues harbored by lumpfish (19). These tissues represent one example of how these animals’ visual system might have adapted to their widely ranging habitat (20). Since our initial study (19), our group has continued to further characterize the eyes of cultured and wild lumpfish at the histological level. We have observed that otherwise healthy wild caught lumpfish without exophthalmos harbor cystic-like structures containing what appears to be leukocyte-like cellular elements in their scleral cartilage. Lumpfish possess scleral skeletal tissue associated with their eye globes similar to those described in other teleosts (21). Pathological cases of chondritis, an inflammatory reaction within cartilage structures, have been previously described in domesticated mammals, experimental animal models and humans (22–24), but we are not aware of previous reports of leukocytic tissue within teleost scleral skeletal tissues.

It has been described that *Myxobolus albi*, a metazoan parasite, infects the scleral cartilage and causes mild to severe exophthalmos in lumpfish (25). In addition, *Vibrio anguillarum*, a Gram-negative bacterium and the causative agent of vibriosis, is a frequent pathogen of lumpfish and causes exophthalmos (26). The rationale of our study was to explore any intraocular immune response in wild lumpfish harboring scleral cysts and cultured lumpfish infected with *V. anguillarum*.

Gross anatomy, histology, immunohistochemistry, genetic and bioinformatic approaches have been utilized to explore the cellular characteristics of scleral cartilage infected with *M. albi* cysts and *V. anguillarum*. Herein, we report here that the intraocular immune response to myxozoan cysts and *V. anguillarum* infection are associated with a host response involving IgM+ cells and CD10+ cells, which, in the context of the immune system, is a marker for hematopoietic cells (27, 28). These results indicate a novel ocular immune system component in response to infection in a teleost.

## Materials and Methods

### Lumpfish Samples, Histology, and Whole Mounts

The work described herein was performed on specimens of lumpfish obtained at the Dr. Joe Brown Aquatic Research Building (JBARB), Department of Ocean Sciences, Memorial University, under approval by the Institutional Animal Care Committee (protocol #17-03-RG). Lumpfish eyes were collected from wild male adults of unknown age and approximately 0.5 kg (n=2), wild female adults of unknown age and approximately 3–4 kg (n=3) and wild female adults that were domesticated for 4 years (n=4) in the JBARB lumpfish culture facility. The wild male animals were collected from Harbour Main of the Newfoundland Avalon Peninsula in 2019. The wild female animals were collected from Newfoundland coastal waters in two different years (two from 2018 from several different geographical locations around the Avalon Peninsula Newfoundland and one from the Champney’s area of Trinity Bay, Newfoundland in 2019). All wild fish were considered as overtly healthy. None showed clinical signs of illness or exophthalmos. Fish were euthanized by a lethal dose of TMS/MS-222 (400 mg/L). Eyes were carefully excised, either fixed in 4% paraformaldehyde and processed for paraffin embedding or freshly dissected for tissue collection and whole mount Alcian blue (cartilage)/Alizarin red (bone) staining. Dissections were performed under a stereomicroscope fitted with an eyepiece adapter facilitating photography.

Paraffin blocks were sectioned and slides stained with hematoxylin and eosin (H&E) or the following special stains. Giemsa stain is commonly utilized to visualize white blood cells, Van Gieson stain is commonly used to detect matrix components such as elastin, Masson’s Trichrome stain is useful for differentially staining elements of bone, cartilage, extracellular matrix and leukocytic features, and basic fuchsin/toluidine blue stain can be used to detect cellular elements (29).

To visualize the lumpfish ocular skeleton, whole mounts of scleral cups or were stained with Alcian blue (to stain cartilage blue) and Alizarin red (to stain ossified bone elements red) as previously described (30). Bony fin tissue from the same animals was used as a positive control tissue. Differential histological stains and whole mount stains were performed by the Histology Core Facility in the Faculty of Medicine, Memorial University.

### Detection of *M. albi* DNA in Lumpfish Scleral Cartilage Tissue

Scleral cartilage cup tissues with or without grossly visible white scleral cartilage tissue pockets were collected from freshly euthanized wild adult lumpfish. Additionally, similar size scleral cartilage cups were collected from cultured lumpfish. Tissues were snap frozen, after which DNA was then isolated from the tissues. Specific primer sets included 538MyxFw and 540MyxRv targeting a 1.5 kb fragment of genomic *M. albi* DNA encoding for the 18S ribosomal RNA (EU420055.2) were generated and used in polymerase chain reaction (PCR) as previously reported (25). Mouse (*Mus musculus*) DNA and naïve cultured lumpfish DNA were used for negative controls.

### Lumpfish Holding, Immunization, and *V. anguillarum* Challenge

*V. anguillarum* infected lumpfish eye tissues were sourced from ongoing vaccination studies. The immunization and challenge experiments were performed in accordance with the guidelines of the Canadian Council on Animal Care and approved by Memorial University of Newfoundland’s Institutional Animal Care Committee (protocols #18-01-JS; #18-02-JS). Juvenile specimens of lumpfish 50 ± 0.2 g (mean ± SE) were obtained from the JBARB at the Department of Ocean Sciences, Memorial University of Newfoundland, Canada. The animals were kept according to established conditions (28), 10°C in 500 l tanks supplied with 95%–110% air saturated and UV treated filtered flow-through seawater, and an ambient photoperiod. The fish were fasted during 48 h pre-vaccination. The fish were intra-peritoneally (IP) vaccinated with formalin inactivated *V. anguillarum* J360 (Chromosome I, NCBI accession number: CP034572.1; chromosome II, NCBI accession number: CP034573.1) and PBS-mock control. After 8 weeks-post vaccination the animals were challenged with *V. anguillarum* J360 (6.7x10^6^ CFU/dose; for fish numbers and vaccinology results see (28). Lumpfish ocular tissues were collected at 7 days post-challenge and processed for paraffin sectioning, histology and CD10 immunostaining described below.

### Lumpfish IgM Purification and Chicken IgY Anti-Lumpfish IgM Production

To assess the presence of lumpfish IgM expression, an anti-lumpfish IgM antibody was produced in chicken. Lumpfish IgM was purified according to protocols described previously for other Teleosts (31) with modifications. Briefly, IgM was purified from pooled lumpfish fresh serum (250 ml) using an immobilized mannan binding protein (MBP) column kit (Pierce Biotechnology) according to manufacturer´s instructions, except that 250 ml of serum were used instead of 1 ml. The integrity and purity of the lumpfish IgM was evaluated by 10% SDS-PAGE (**Figure S1**). Chicken IgY anti-lumpfish IgM antibody was produced and biotinylated commercially at Somru BioScience Inc. (Charlottetown, PEI, Canada). Briefly, chickens were immunized *via* intramuscular injection into breast muscle with a maximum volume of 500 µl/injection and a maximum immunogen concentration of 0.4 mg/ml (200 µg/500 µl). Two chickens were immunized with lumpfish IgM in combination with Freund’s complete and incomplete adjuvant. The immunization series was initiated when chickens were 14 weeks of age. The initial immunization was 400 µg of lumpfish IgM in Freund’s Complete adjuvant in a total volume of 1 ml. Four subsequent immunizations of 200 ug of lumpfish IgM in Freund’s incomplete adjuvant in a total volume of 1 ml were delivered at 14 day intervals. For all immunizations, 0.5 ml of immunogen preparation was delivered per breast muscle. Serum titers were monitored every 14 days using direct ELISA. Western blot of purified lumpfish IgM was performed using the chicken IgY anti-lumpfish IgM primary antibody and goat anti-IgY-HRP (horseradish peroxidase; Promega) secondary antibody (**Figure S1**).

### Immunohistochemistry

Immunohistochemistry (IHC) for CD10 was performed using a Ventana Benchmark Ultra automated immunostainer (Roche) in the Department of Anatomical Pathology, General Hospital, Eastern Health, St. John’s NL, on sections from lumpfish eyes, lumpfish head kidney and human tonsil applied to positively charged slides using rabbit monoclonal IgG antibody clone SP67 directed against human CD10. Sections were processed on the automated immunostainer using citrate-based (10 mM, pH 6) and tris-based buffer CC1 (Roche, Switzerland) for antigen retrieval at 100°C for 64 min followed by 32 min of incubation at room temperature with either rabbit monoclonal IgG antibody clone SP67 anti-human CD10 (Roche, Diagnostic 790-4506) or a rabbit IgG (Roche, Diagnostics 790-4795) as negative control using a 1:200 dilution and detected using Ultraview (Roche) and counterstained with hematoxylin. Anti-alpha smooth muscle actin (ASMA) IHC was performed as previously described (19). Double anti-CD10/ASMA IHC was performed on a Leica Bond automated immunostainer using parameters similar to those used for automated CD10 staining. For IgM IHC, we used the anti-lumpfish-IgM IgY antibody custom produced in collaboration with Somru BioScience described above. Anti-lumpfish-IgM IgY was applied at a 1/500 dilution of a 2.7 mg/ml stock concentration using IHC procedures previously described (19) except that an alkaline phosphatase-conjugated anti-IgY secondary antibody was used at 1/250 dilution to develop the IgM staining reaction. As a specificity control, the anti-lumpfish-IgM IgY antibody was pre-absorbed for 1 h with a 100-fold excess concentration of purified lumpfish-IgM prior to being applied to the sections for IHC (32).

### Sequence Analysis

Nucleotide Basic Local Alignment Search Tool (BLAST) 2.7.1 (33) was performed based on the sequences of the putative lumpfish CD10 present in the public genome sequence of *C. lumpus* (34), and *Homo sapiens* CD10 (EAW78758.1) gene sequence. Amino acid sequence alignments were performed using the CLC Workbench 20 (Qiagen). For phylogenic analysis, the bootstrap consensus tree inferred from 100 replicates were taken to represent the evolutionary history of the taxa analyzed (35). Branches corresponding to partitions reproduced in less than 50% bootstrap replicates were collapsed. Initial tree(s) for the heuristic search were obtained automatically by applying Neighbor-Joining and BioNJ algorithms to a matrix of pairwise distances estimated using a JTT model, and then selecting the topology with superior log likelihood value. The analysis involved 34 amino acid sequences. All positions containing gaps and missing data were eliminated. There was a total of 704 positions in the final dataset. Evolutionary analyses were conducted in MEGA7 (36). Protein structural-based alignments were performed by using the web-based interface for ESPript v.2.2 located at http://espript.ibcp.fr/ESPript/cgi-bin/ESPript.cgi (37). The 3D structure of *C. lumpus* CD10 protein was predicted using position specific iterative-BLAST (PSI-BLAST) alignment and HHpred (https://toolkit.tuebingen.mpg.de/#/) (38, 39).

## Results

### Gross Anatomical and Histological Characteristics of the Scleral Skeleton of Wild Lumpfish in the Context of Myxozoan Cysts

**Figure 1:**
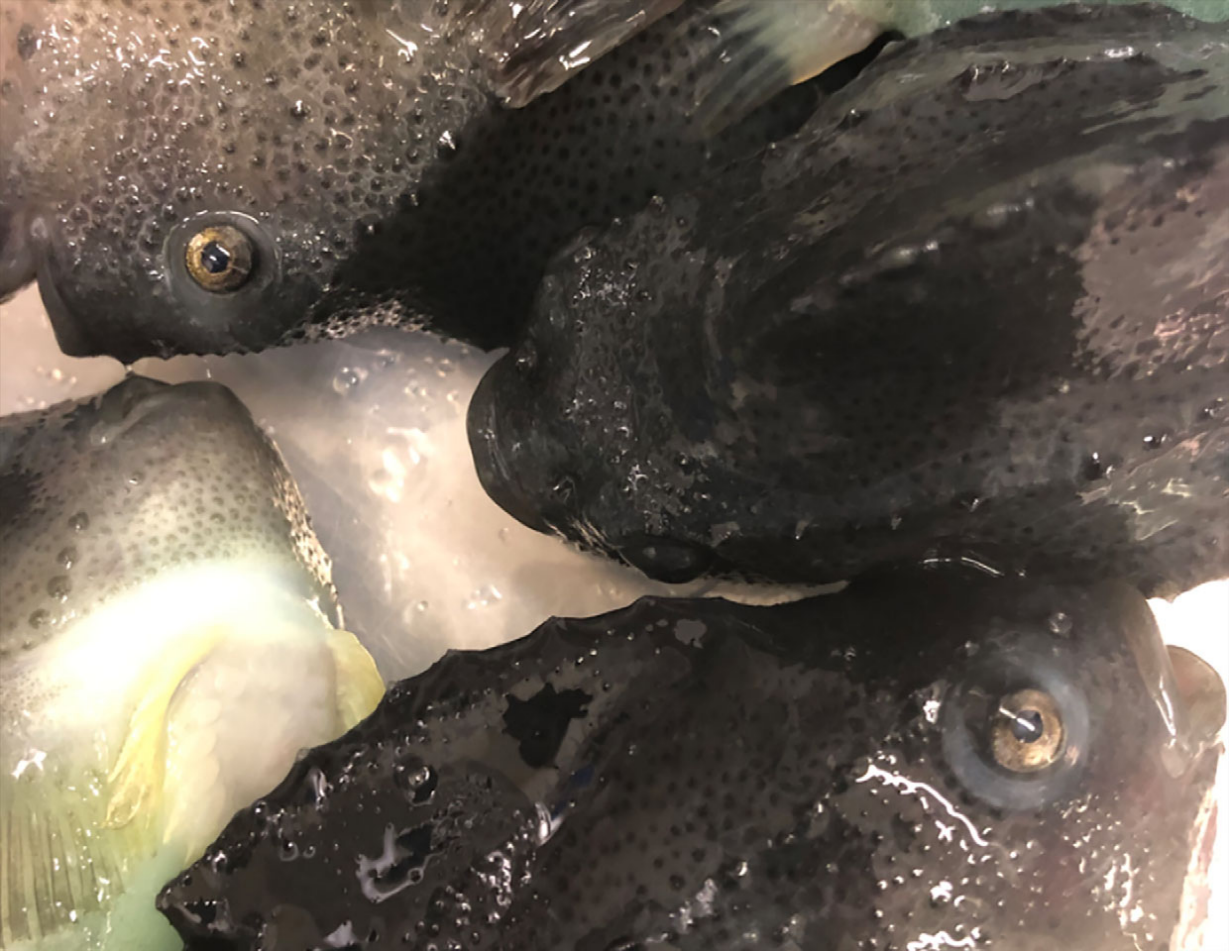
Normal wild adult lumpfish. Head and eye regions of several approximately 3 kg wild adult female North Atlantic lumpfish, representative of most of those studied herein, are shown immediately following sacrifice. As exemplified in the image, the wild specimens studied herein appeared overtly healthy and did not show any overt signs of clinical eye disease or exophthalmos.

**Figure 2:**
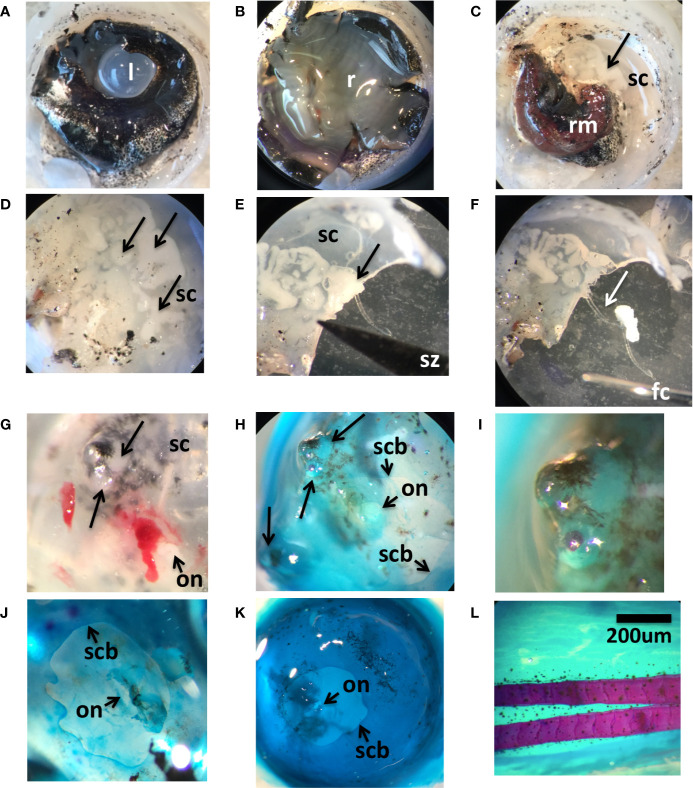
Anatomical characteristics of wild adult lumpfish scleral cartilage. Panels **(A–F)** show an anterior-posterior sequential dissection of an eye from a ~3 kg North Atlantic wild adult lumpfish revealing, first, lens (l) set in middle of pigmented iris **(A)**, next, retina (r) **(B)**, next, the *rete mirabile* vascular organ (rm) **(C)** and finally the semi-transparent scleral cartilage (sc) **(C–E)**; arrows show the cysts. Panel **(E)** shows scleral cartilage cut in half through a cystic structure (arrow) with the tip of the scissors (sz) visible in the image. Panel **(F)** shows the soft white clump of tissue (arrow) pulled out of the scleral pocket with ophthalmologic forceps (fc) of which one metal tip is visible in the image. Panels **(G–I)** show an eye from a ~0.5 kg adult North Atlantic wild adult lumpfish. Panel **(G)** shows freshly dissected scleral cartilage (sc) with protruding lesions (large arrows) next to the optic nerve head (on). Panels **(H, I)** show the same region after Alcian blue/Alizarin red whole mount staining, panel **(I)** magnified twice as much. Alcian blue/Alizarin red staining of scleral cup from another ~0.5 kg adult North Atlantic wild adult lumpfish **(J)** and from a similar size adult North Atlantic cultured lumpfish **(K)**. In panels **(J, K)**, the boundary of the scleral cartilage and sclera are indicated (scb), as is the position of the optic nerve head (on). Panels **(G–K)** are from an ocular posterior viewpoint. Panel **(L)** shows fin tissue from one of the same adult North Atlantic cultured lumpfishes, stained with Alcian blue/Alizarin red, and which was used as a positive control for fully ossified bone tissue as seen by red fin bone staining. Dissected and whole mounted eyes were previously fixed in 4% paraformaldehyde which slightly opacifies the lens. Magnification: 65X. Scale bar in **(L)** applies to all panels.

**Figure 3:**
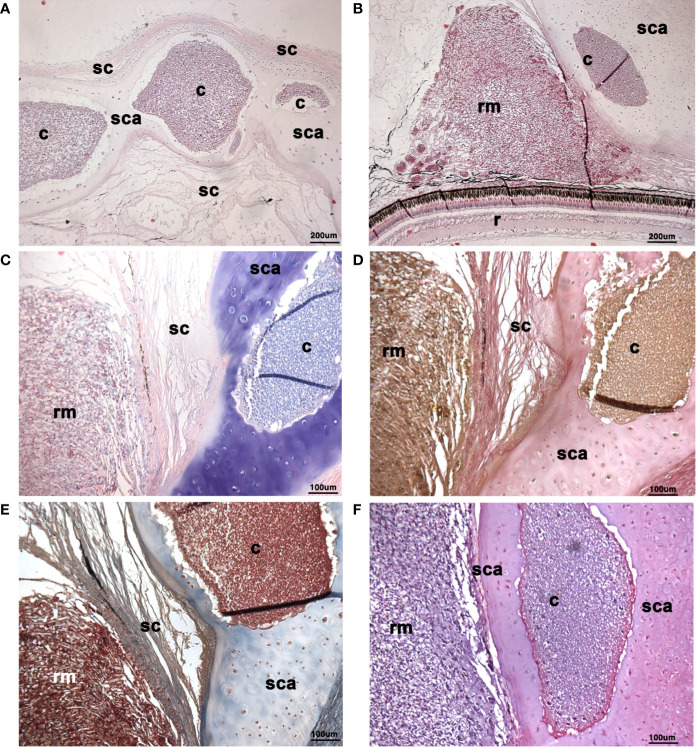
Histological morphology of cysts in scleral cartilage of wild adult lumpfish eyes. Histology of wild lumpfish eye displays extensive cystic structures containing densely packed cellular tissue present in spaces of the scleral cartilage when stained with Hematoxylin & eosin **(A, B)**, Giemsa **(C)**, Van Gieson **(D)**, Masson’s Trichrome **(E)**, and basic fuchsin/toluidine blue **(F)**. The scleral cartilage cysts were located in central portions of the eye globe, in close proximity to the most robust portions of the *rete mirabile* vascular tissue (rm). Consistent with the differential staining utility of Giemsa staining **(C)** which differentially stains white blood cells (blue) versus red blood cells (pink), the scleral cartilage cysts stained mainly blue with Giemsa versus the more pink Giemsa staining observed in the *rete mirabile* which is rich in red blood cells. Van Gieson **(D)**, which stains all blood cells gold, and basic fuchsin/toluidine blue **(F)** which stains all blood cells purple, revealed a histological staining pattern for the scleral cartilage cysts that was similar to the blood cell elements in the *rete mirabile* (rm). The scleral cartilage matrix material stained a typical blueish green with Masson’s Trichrome while the cysts contained highly eosinophilic staining **(E)**. r, retina; rm, *rete mirabile*; scl, sclera; sca, scleral cartilage; c, cyst. Magnification: **(A, B)** 100X; **(C–F)** 200X. Representative images are shown.

All of the wild lumpfish studied herein were otherwise overtly healthy showing no signs of illness or exophthalmos (**Figure 1**). We observed, histologically, pockets of densely packed cellular tissue within their eye scleral cartilage (described below in **Figure 3**). Such structures were similar to myxozoan cysts previously described in diseased aquaria lumpfish (25). Sequential anterior to posterior anatomical dissection of the lens, retina, and *rete mirabile* vascular tissues (40) of wild adult lumpfish eye revealed the cysts in the semi-transparent scleral skeletal tissue (**Figures 2A–E**). Soft clumps of cystic material can be easily pulled out from the scleral skeletal tissue with ophthalmologic forceps (**Figure 2F**) and were confirmed to be myxozoan positive (see 3.2).

These cysts in scleral cartilage were seen in three out of five independent healthy wild specimens. Two of these specimens were harvested in 2018 and one in 2019 from separate geographical locations near St. John’s NL. We did not observe scleral cartilage cysts in any of 4 wild lumpfish specimens of comparable size that were domesticated in the JBARB lumpfish culture facility for 4 years.

Further characterization of the ocular cysts was performed. Whole mount staining with Alcian blue and Alizarin red, of the scleral cups from two of the healthy wild caught adult lumpfish, revealed that one animal harbored grossly visible, posteriorly protruding, scleral skeletal cysts clearly embedded within the blue staining scleral skeletal tissue, resembling tubercles (**Figures 2G–I**). Alcian blue cartilage staining displayed the expected general shape and configuration of the scleral cartilage cup, including a posterior window of non-cartilaginous, non-ossified sclera to accommodate the optic nerve and *rete mirabile* tracts (**Figure 2J**).

Whole mount staining of a scleral cup from a cultured lumpfish specimen revealed no scleral cartilage cysts but did show the same general shape and configuration of the scleral cartilage cup with posterior scleral optic tract window as with the wild specimen (**Figure 2K**). Absence of Alizarin Red staining in either the wild or the cultured lumpfish scleral cups indicated no ossification to bone tissue, as compared with the positive control fin (see red staining tissue in **Figure 2L**).

**Figure 4:**
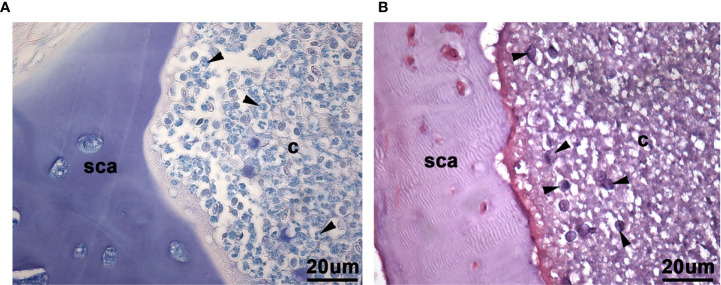
Higher power view of leukocyte-like cells and myxozoan spores in Giemsa and basic fuchsin/toluidine blue histological stains of scleral cartilage cysts of wild adult lumpfish eyes. Giemsa **(A)** which stains blood cell nuclei deep blue, revealed a polymorphonuclear histological staining pattern of some cells (arrowheads in **(A)**) reminiscent of granulocytes in the scleral cartilage cysts. Basic fuchsin/toluidine blue stains **(B)** revealed myxozoan spores with or without polar bodies, depending on plane of section (arrowheads in **(B)**). sca, scleral cartilage; c, cyst. Magnification: 400X. Representative images are shown.

Histological sections of the otherwise healthy wild adult lumpfish eyes displayed extensive densely packed cystic structures with what appeared to be myxozoan spores (25) present in the scleral cartilage (**Figures 3A–F** and **4A, B**). Histologically, the cystic tissue was strikingly similarity to mammalian active bone marrow, a tissue containing high numbers of leukocytes and little or no fat tissue (29), but also contained hallmarks of scleral myxozoan cysts with spores previously described as being associated with exophthalmos in *M. albi* infection in scleral cartilage of clinically ill captive lumpfish (25).

The scleral cartilage cystic structures were found in central portions of the eye globe, in close proximity to the most robust portions of the *rete mirabile* (as shown in **Figures 2** and **3A–F**). Giemsa stain, which differentially stains most leukocytes blue and erythrocytes pink, revealed small blue staining white blood cell-like cells with basophilic nuclei and larger blue staining stromal-like cells in the cysts of the scleral cartilage (**Figures 3C** and **4A**). Giemsa staining of the *rete mirabile* revealed a more prominent pink staining than the scleral cartilage cysts by virtue of the larger proportion of red blood cells within the *rete mirabile* (**Figure 3C**).

Van Gieson stain which is classically used to define matrix components such as elastin staining all blood cells gold, revealed a staining pattern of the scleral cartilage cysts similar to the blood cell elements in the *rete mirabile* (**Figure 3D**). The scleral cartilage matrix material stained a typical blueish green color with Masson’s Trichrome (blue staining of bone, cartilage and extracellular matrix and eosin staining of granulated cells), while the leukocytic-like elements stained red similarly to the blood cells in the *rete mirabile* (**Figure 3E**). Both H&E and basic fuchsin/toluidine blue stains also revealed a similar staining pattern for the blood cell-like elements of the scleral cartilage cysts and the blood cells in the *rete mirabile* (**Figures 3B, F**).

**Figure 5:**
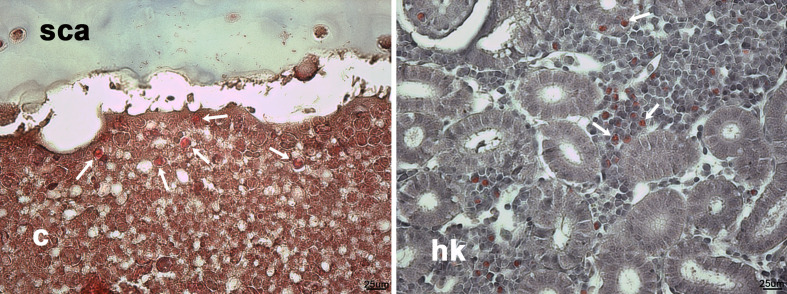
Eosinophilic leukocyte-like cells in Masson’s trichrome histological stains of scleral cartilage cysts of wild adult lumpfish eyes. Masson’s trichrome (left) revealed intense eosinophilia of some cyst cells (arrows), similar to eosinophilic cells (arrows in right) in leukocyte rich regions of head kidney. c, cyst; hk, head kidney. Magnification: 400X. Representative images are shown.

Closer examination of the basic fuchsin/toluidine blue stain indicated the presence of the myxozoan spores, and, in some cases at different planes of section, revealing the spore polar bodies previously described for the *M. albi genus of* these myxozoans (25), (**Figure 4B**). The Masson’s trichrome stain of the scleral cartilage cysts also revealed, at high magnification (400x), highly eosinophilic cells similar to those which were also present in leukocytic rich regions of head kidney (**Figure 5**).

The presence of lymphocytic-like cells (small round cells with a large nucleus and little cytoplasm) and larger cells were observed in tissue stained with Giemsa or basic fuchsin / toluidine blue differential histological staining (**Figure 4**). These results suggest that at least some of the cells in the scleral cartilage cysts could be leukocytes.

The scleral cartilage cysts do not appear to be lined by perichondrium, a connective tissue layer that overlies surfaces of normally developing scleral cartilage tissue in teleosts (41). Although a thin but histologically distinct layer of material is observable lining the cysts in specimens stained with Masson’s trichrome (**Figure 3E**) and basic fuchsin/toluidine blue (**Figures 3F** and **4**), it did not appear to contain nucleated cells and therefore does not resemble true perichondrium.

### Myxozoan Presence in Wild Lumpfish Scleral Cartilage

**Figure 6:**
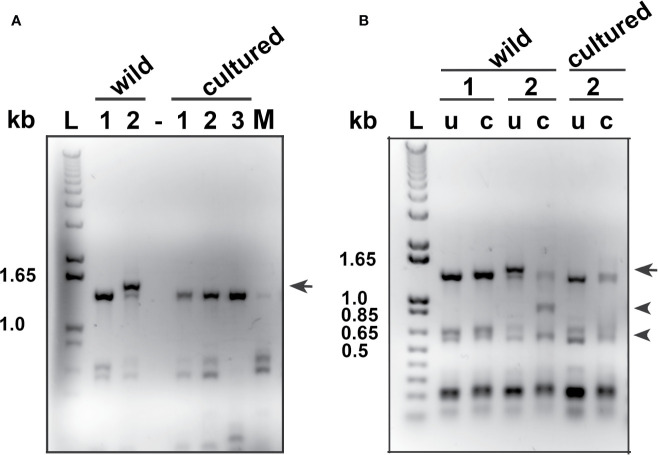
Polymerase chain reaction (PCR) analysis of *M. albi* 18S ribosomal DNA in lumpfish specimens. **(A)** Representatives PCR analyses for *M. albi* 18S ribosomal DNA of wild lumpfish specimens with (wild #2 lane) and without (wild #1 lane) white scleral cartilage cysts revealed amplification of an expected 1.5 kb DNA fragment (arrow) only in the wild specimen with visible scleral cartilage cysts. The 1.5 kb DNA amplicon was not detected in PCR analyses for *M. albi* 18S ribosomal DNA of scleral cartilage cup tissues collected from freshly euthanized, similarly sized cultured lumpfish (cultured #1, 2 and 3 lanes) or, of DNA samples prepared from mouse tissues (M lane) and in the absence of DNA (lane -). **(B)** BglII digestion of PCR reactions for *M. albi* 18S ribosomal DNA (lanes c) resulted in the cut of the 1.5 kb amplicon into the expected two fragments of ~910 bp and ~605 bp (arrowheads: wild #2 lane, c). These two fragments were not detected in BglII digests of PCR reactions from lumpfish without visible scleral cartilage cysts (wild #1 lane and cultured #2 lane). Lanes u: uncut PCR reactions; lane L: 1 kb plus DNA ladder.

PCR analyses for *M. albi* 18S ribosomal DNA of wild lumpfish specimens with scleral cartilage cysts revealed amplification of the expected 1.5 kb DNA fragment (**Figure 6A**). The 1.5 kb DNA amplicon was not detected in PCR analyses for *M. albi* 18S ribosomal DNA of scleral cartilage cup tissues collected from freshly euthanized, similarly sized cultured lumpfish, or of DNA samples prepared from mouse tissues (**Figure 6A**). Restriction digests of the PCR reaction amplicons with *Bgl*II were next performed to verify the specificity of the 1.5 kb amplicon for *M. albi* 18S ribosomal DNA (**Figure 6B**). *Bgl*II digestion resulted in digestion of the 1.5 kb amplicon into the expected two fragments of ~910 bp and ~605 bp (**Figure 6B**).

### IgM Expression in Wild Lumpfish Scleral Cartilage Cysts

**Figure 7:**
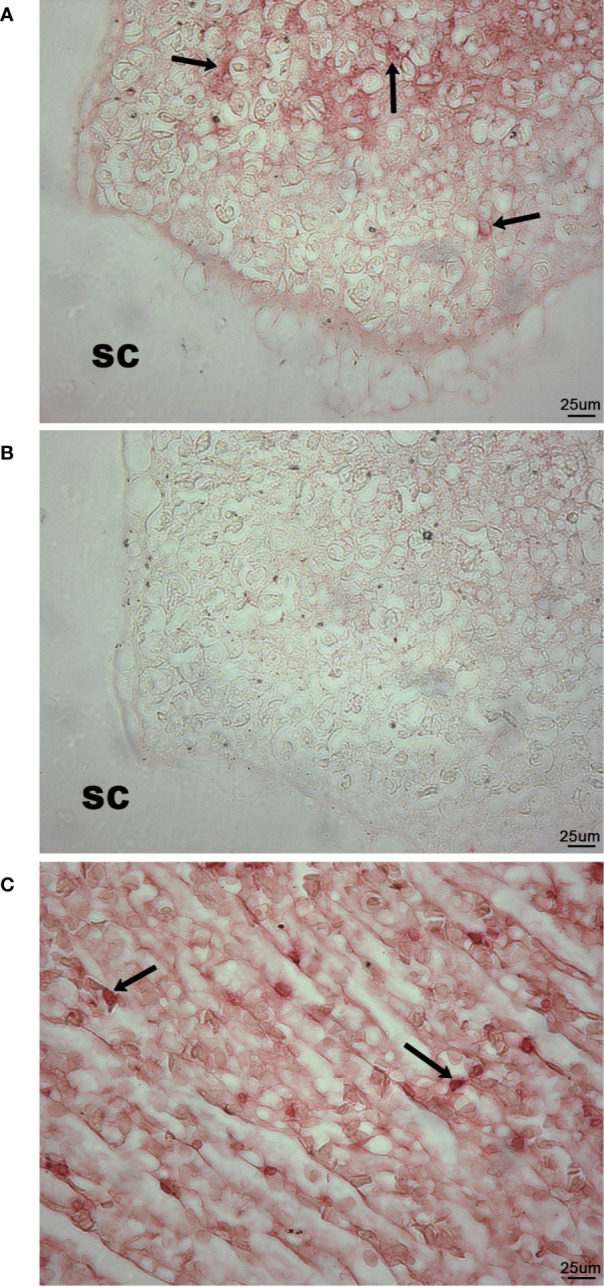
Immunohistochemistry for IgM in wild adult lumpfish eyes. IgM staining in scleral cartilage cysts **(A)** and blood vessels of the *rete mirabile* **(C)** appeared as a bright red staining reaction. No counterstain was applied in order to emphasize both the densely focused cellular IgM and the sometimes diffusely distributed IgM expression. IgM expression was observed within blood vessels of the *rete mirabile* (used as positive control) **(C)** but not in the cartilaginous portions (sc) of the scleral cartilage **(A)**. Arrows in **(A, C)** indicate examples of individual cells expressing high levels of IgM. Further negative control using the anti-lumpfish IgM IgY antibody pre-absorbed for 1 h with a 100-fold excess concentration of purified lumpfish IgM resulted in no staining of the scleral cartilage cyst **(B)**. Magnification: 400X; scale bars 50 um. We observed scleral cartilage cysts harboring IgM^+^ cells in scleral cartilage of three out of five independent healthy wild specimens. Representative images are shown.

Histological staining suggested that the scleral cartilage cysts contained cells of the immune system. High levels of expression of IgM (42, 43) were detected by IHC mainly in the center of the scleral cartilage cysts while more diffuse lower levels were observed at the edges of the cysts. Mainly diffuse, lower levels of IgM staining with occasional densely focused expression pattern on individual cells was also present within the lumens of blood vessels of the *rete mirabile* as expected, serving as a positive internal control for IgM staining (**Figure 7C**). IgM expression was not observed in neural retinal tissue (not shown) or in the cartilage tissue surrounding the scleral cartilage cysts (**Figure 7A**). Furthermore, using the anti-lumpfish IgM IgY antibody pre-absorbed for 1 h with a 100-fold excess concentration of purified lumpfish IgM resulted in no staining of the scleral cartilage tissue (**Figure 7B**). Western blot of purified lumpfish IgM further confirmed the specificity of the anti-lumpfish IgM antibody for the 75 kDa heavy and 25 kDa light chains of lumpfish IgM and for the various dimers formed by these chains (**Figure S1**).

### CD10 Expression in Normal Lumpfish Tissues

**Figure 8:**
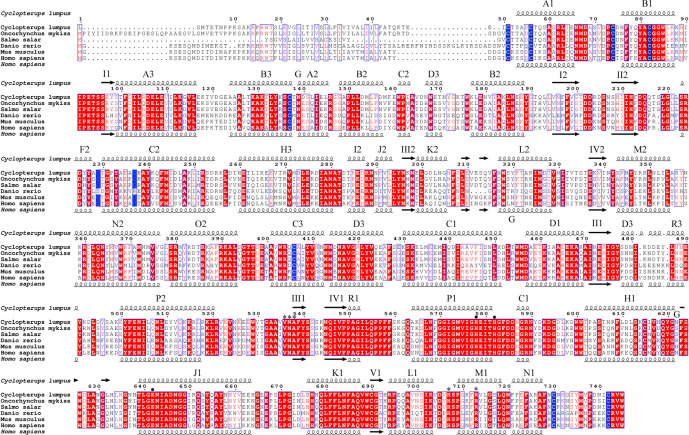
Lumpfish CD10 alignment and secondary protein structure. The blue boxed regions indicate the conserved cysteine residues that form a disulfide-bound partner. The black dots indicate the essential residues for proteolytic activity. The asterisk regions (*) indicate the ligand binding-residues.

**Figure 9:**
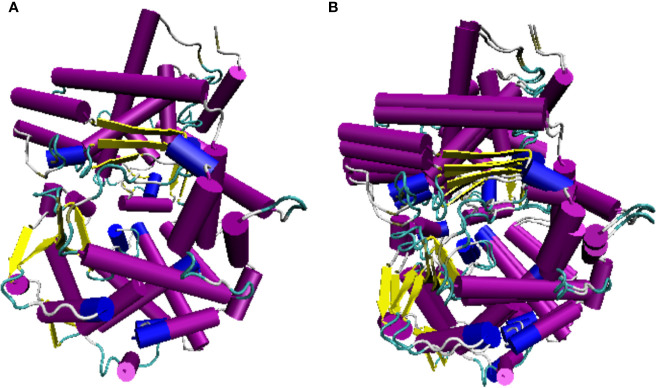
Lumpfish CD10 3D predicted protein structure. **(A)** Lumpfish CD10 protein, **(B)** Lumpfish and human CD10 protein overlap. The purple and blue cylinders represent α-helix structures and the yellow arrows represent the β-sheet structures. *H. sapiens* CD10 accession number: NP_000893.2; *C. lumpus* CD10 accession numbers: MT978157, MT978158, MT978159, MT978160, MT978161, MT978162, MT978163, and MT978164.

In addition to IgM, we searched for potential molecular homologs expressed in cells of the mammalian immune system to confirm the leukocyte identity of the cyst cells. CD10 is a cell surface endopeptidase expressed in cells involved in the immune response in mammals (44). We found that the sequence of human CD10 protein is overall 60% identical with the putative lumpfish CD10 protein sequence (**Figure 8**). The secondary structure domains, including the conserved cysteine residues that form a disulfide-bound partner, the essential residues for proteolytic activity and the ligand binding-residues are all identical between lumpfish and *H. sapiens* CD10 (**Figure 8**) (45). Although the protein sequence of lumpfish CD10 presented in **Figure 8** requires further verification, our analysis showed a highly conserved neprilysin active site (45) required for proteolytic activity (**Figure 8**). In addition, the essential residues for protein folding and functional conformation were found conserved in lumpfish CD10 (**Figures 8** and **9A, B**).

**Figure 10:**
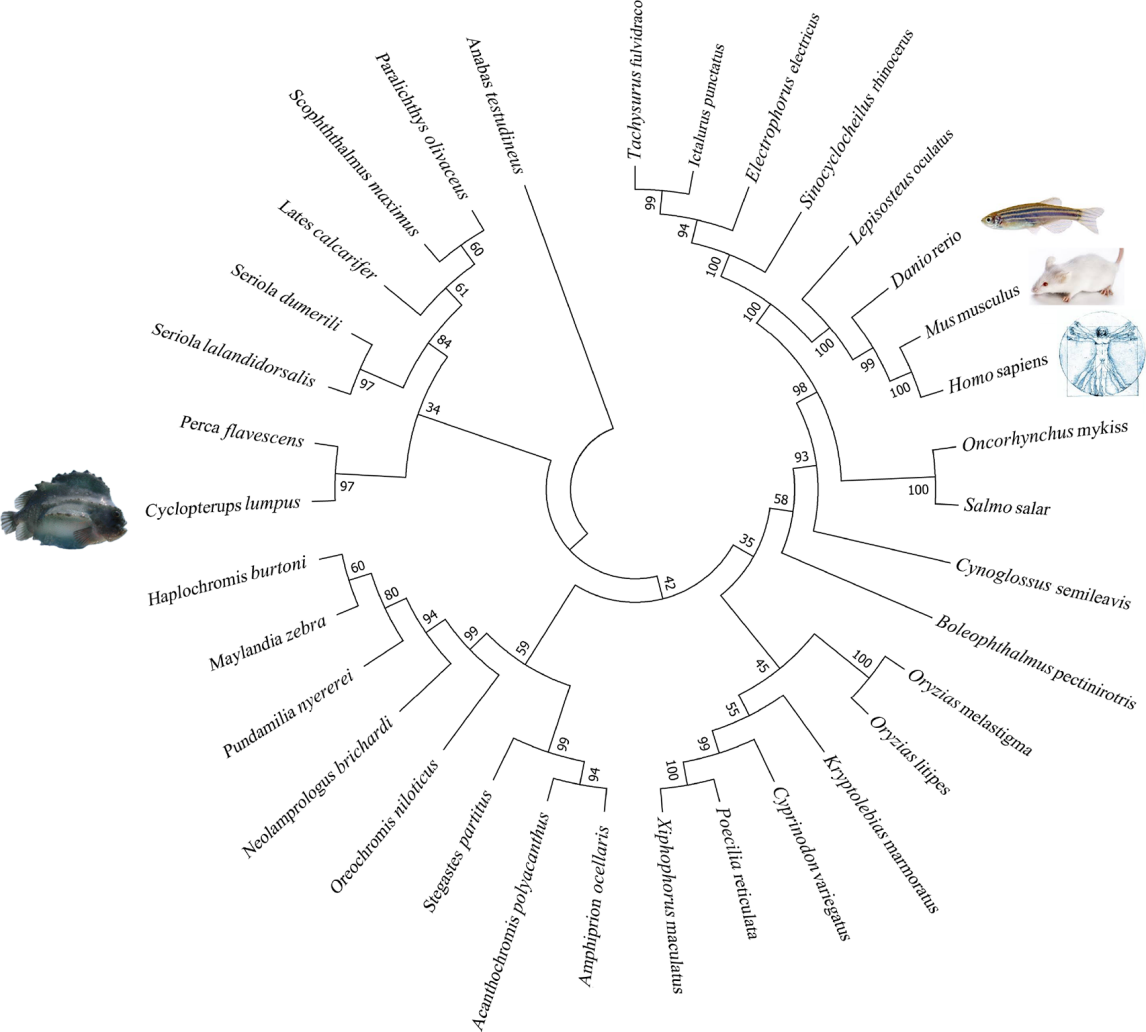
Lumpfish CD10 molecular phylogenetic analysis. The evolutionary history of CD10 was inferred by using the Maximum Likelihood method based on the JTT matrix-based model (46).

The lumpfish CD10 structural prediction analysis supported the presence of the residues required for correct folding of the protein (**Figures 9A, B**). Two of the three glycosylation sites present in human CD10 were found present in lumpfish CD10. However, these glycosylation sites are not well conserved in the sequences of other vertebrate CD10 (**Figure 8**). Phylogenetic analysis of lumpfish CD10 revealed that it is closely related to other Teleosts but distantly related to *Danio rerio*, *Mus musculus*, and *Homo sapiens* (**Figure 10**).

**Figure 11:**
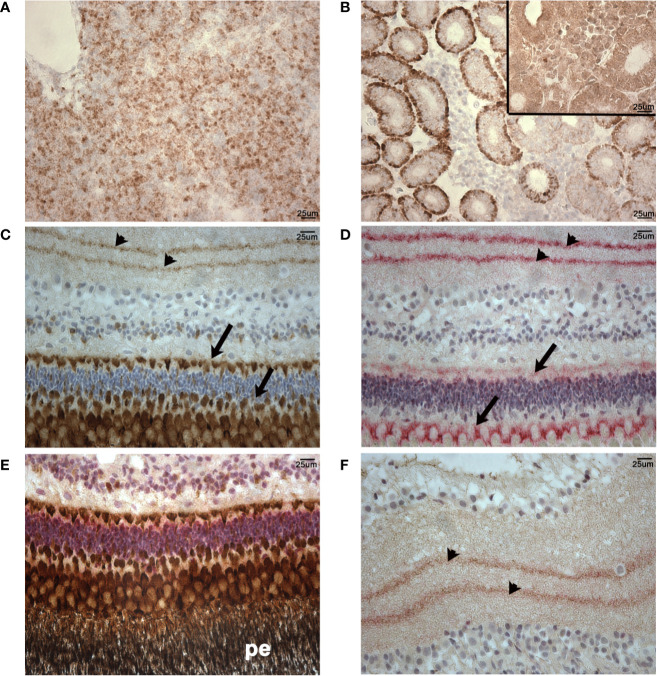
Normal patterns of CD10 and ASMA expression in lumpfish tissues. CD10 expression (brown staining) was detected in numerous small cells in follicular regions of the head kidney **(A)** and basal cells of the renal tubules **(B)**. Inset in **(B)** is a negative control of an adjacent section of those used in **(A, B)** stained using no primary antibody. **(C)**, CD10 expression (brown staining) was detected in a well-defined peri-photoreceptor pattern in the outer layers of the retina (arrows) and in well-defined bands in the inner layers of the retina (arrowheads). **(D)**, ASMA expression (red staining) pattern in the retina was highly similar to CD10 pattern of expression. CD10 and ASMA double staining in peri-photoreceptor pattern in the outer layers of the retina **(E)** and inner layers of the retina **(F)**. The pigmented epithelial layer (pe) in panel **(E)** is black due to the endogenous pigment in these cells. Double stained sections show overlap of stain and a blended magenta staining color in areas of expression overlap (arrowheads in **(F)**). Magnification: 400X. Scale bars in **(A, B)** are also representative of scale in **(C–F)**. Representative images are shown.

We utilized a rabbit anti-human CD10 monoclonal antibody (validated using clinical reference standard at NordiQC https://www.nordiqc.org/epitope.php?id=23), raised against amino acids 450–550 of human CD10 which, as shown in **Figure 8**, has 68% identical matches and 80% positive matches with the putative lumpfish CD10 protein sequence. In addition, amino acids 450–550 of the human CD10 protein sequence share 22 regions of 100% identity with the lumpfish CD10 orthologue. Moreover, we have recently successfully utilized this anti-CD10 antibody to stain CD10 positive cells in an experimental infection scenario in various tissues of other teleosts (47). Using this antibody we identified specific and distinctive CD10 expression in lumpfish head kidney, a tissue rich in lymphoid cells (48), and in the lumpfish eye (**Figure 11**). CD10 was expressed in numerous small leukocytic-like cells in follicular regions of the head kidney and in basal cells of the renal tubules of the head kidney (**Figures 11A, B**). We also found CD10 expression in a well-defined peri-photoreceptor pattern in the outer layers of the retina and in well defined bands in the inner layers of the retina (**Figures 11C, D**), both of which largely colocalize with expression of alpha smooth muscle actin (**Figures 11E, F**) (ASMA), a marker we have previously described to be expressed in lumpfish retina (19).

### CD10 Expression in Wild Lumpfish Scleral Cartilage Cysts

**Figure 12:**
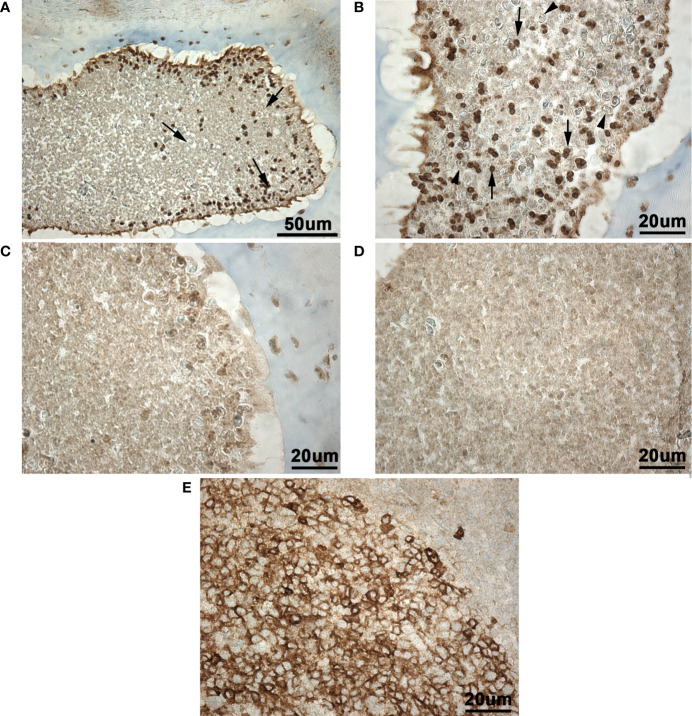
Immunohistochemistry for CD10 in wild adult lumpfish scleral cartilage cysts. CD10 stain of cysts present in the scleral cartilage appeared as dark brown staining [arrows in **(A, B)**] against light brown background staining seen in **(C, D)** and a light blue hematoxylin counterstain. Sections adjacent to those used for anti-CD10 staining but instead stained with negative control [rabbit IgG **(C, D)**] show a similar level of background staining reaction to that seen in the background of the anti-CD10 preparations. The cysts present in the scleral cartilage contained numerous small round leukocyte-like cells expressing CD10 well above the background staining seen in **(C, D)**. In higher power view **(B)**, polymorphonuclear cells reminiscent of granulocytes and negative for CD10 were observed in the scleral cartilage cysts (arrowheads). **(E)**, Positive control human tonsil section serves as a positive staining control for CD10 (dark brown staining). Magnification: **(A)**, 200X; **(B-E)**, 400X. We observed scleral cartilage cysts harboring CD10^+^ cells in scleral cartilage of three out of five independent healthy wild specimens. Representative images are shown.

Immunohistochemistry revealed the presence of a CD10^+^ cell population in the scleral cartilage cysts of wild adult lumpfish eyes (**Figures 12A, B**). The CD10^+^ cells in the scleral cartilage cysts were relatively small round cells and expressed CD10 intensely. CD10^+^ cells were infrequently observed within blood spaces of the eye (*rete mirabile*, choroidal blood vessels and other vascular compartments) encompassing ocular peripheral blood compartment (not shown). The CD10^+^ cells were located in more peripheral or cortical regions of the scleral cartilage cysts (**Figure 12A**). Negative controls, in which the anti-CD10 primary antibody was substituted with a species-matched IgG antibody using the same CD10 staining protocol, showed only light brown background (**Figures 12C, D**). Human tonsil, a lymphoid tissue, was used as a positive control and this tissue showed numerous CD10^+^ leukocytic cells as expected (**Figure 12E**).

### CD10 Expression in *V. anguillarum* Challenged Lumpfish Ocular Tissue

**Figure 13:**
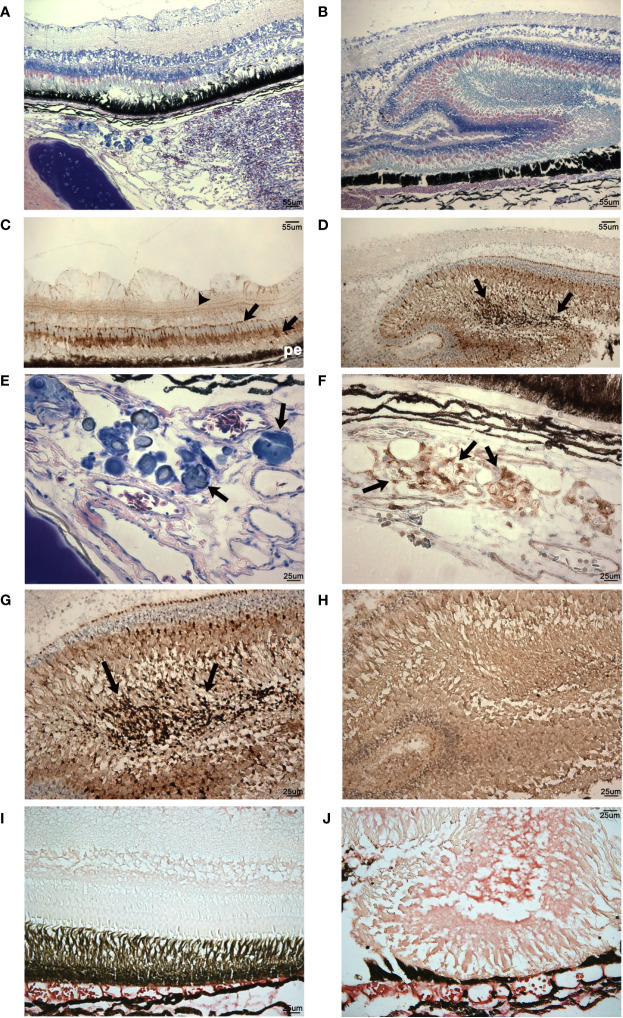
Analysis of histomorphology and CD10 expression in vaccinated and nonvaccinated *V. anguillarum* challenged lumpfish ocular tissues. **(A, B)**, Giemsa staining of retinal regions of vaccinated **(A)** and nonvaccinated **(B)** *V. anguillarum* challenged lumpfish ocular tissues. **(A)**, in the vaccinated challenged animal the scleral cartilage (sc) visible in the lower left corner of the image (dark blue staining), the *rete mirabile* (rm) vascular elements visible in the bottom right quadrant of image and the retinal layers (r) located in the top half of image all appeared relatively undisturbed. **(B)**, in the nonvaccinated challenged animal, the normal retinal layers were lost, retinal tissue, which took up most of the image area, became thickened and disorganized. **(C, D, F**, **G)**, CD10 expression (dark brown) in retinal regions of vaccinated (C: 100X, F: 400X) and nonvaccinated (D: 100X, G: 400X) *V. anguillarum* challenged lumpfish ocular tissues. **(E)**, magnified view (400X) of a subretinal region of the Giemsa staining shown in **(A)** of a vaccinated *V. anguillarum* challenged lumpfish displaying areas of putative chondrogenesis staining dark blue (arrows). **(F)**, CD10 expression (dark brown, arrows) in a section adjacent to that shown in **(E)** of the same vaccinated *V. anguillarum* challenged lumpfish revealed areas of CD10 expression closely coinciding with the areas of putative chondrogenesis visible in **(E)**. Arrows in **(C, D, F, G)** indicate the CD10 stain appeared as deep dark brown staining reaction against the lighter brown background. Sections adjacent to those used for anti-CD10 staining but instead stained with no primary antibody **(H)** show only a similar level of background staining reaction to that seen in the background of the anti-CD10 preparations. IgM expression (bright red stain) is elevated in thickened retinal regions of nonvaccinated animals **(J)** but only in choroidal vasculature (cv) areas of vaccinated animals **(I)** *V. anguillarum* challenged lumpfish. r, retina; rm, *rete mirabile*; sc, scleral cartilage; cv, choroidal vasculature; pe, pigmented epithelium. Magnification: (**A–D**, 100X), **(E–H)**, 400X. Scale bars in **(A, B)** are also representative of scale in **(C, D)**. For these studies, three individual previously vaccinated *V. anguillarum* challenged lumpfish and 4 individual non-vaccinated *V. anguillarum* challenged lumpfish were assessed. Representative images are shown.

In order to assess the role of lumpfish ocular CD10 expression in a separate pathogen infection scenario, histomorphology, CD10 and IgM expression were analyzed in ocular tissues of lumpfish infected with *V. anguillarum*, in vaccinated and naïve lumpfish (non-vaccinated fish). Giemsa staining revealed that the scleral cartilage, the *rete mirabile* vascular elements and the retinal layers, were relatively undisturbed in vaccinated and challenged animals (**Figure 13A**). CD10 expression (**Figure 13C**) was localized mainly in the distinctive retinal banding pattern found in healthy animals as shown in **Figure 11**.

Naïve infected lumpfish developed exophthalmos, and showed various levels of retinal and subretinal tissue disorganization (see lesion portions of retinal tissue arrowed in **Figure 13D** compared to the surrounding, less disturbed retinal tissues visible in **Figures 13B, D**). Most of the naïve infected lumpfish were severely affected, retinal layers structure was lost and retinal tissue became thickened and disorganized (**Figures 13B, E**). CD10^+^ cells were highly present in tissue severely damaged and disorganized (**Figures 13D, F, G**). Patches of what appears to be chondrogenesis were present in one of naïve infected lumpfish (**Figure 13E**). Tissue areas with high density of CD10+ cells closely coincided with these areas of putative chondrogenesis (**Figure 13F**). Also, intraretinal IgM expression was present at high levels in the choroidal vasculature and in specific layers in both thickened lesioned retinal areas (**Figure 13J**) and non-thickened areas of retinal tissues of all naïve infected lumpfish.

## Discussion

Teleosts use soft tissue organs such as head kidney for hematopoiesis (48–50). Since CD10 is known as a marker for hematopoiesis progenitor and stem cells (44, 51), the presence of CD10 cells in ocular tissue raises the possibility that wild lumpfish might utilize a form of ocular extramedullary hematopoiesis, which has been described in mammals (52, 53) for specific adaptations such as during response to ocular infection by pathogens. Teleosts are known to utilize plasmablast and plasma cell derived IgM in response to pathogens (43) and IgM has been demonstrated to play a role in response to inflammatory reaction in lumpfish (54).

Molecular biological studies of species such as lumpfish are impeded by the lack of genetics and antibody reagents specific to the organism under study. Although we were fortunate to be able to assess the expression of lumpfish IgM, among the several antibody reagents raised against mammalian leukocytic markers (CD3, CD45, CD34) that we tested against the scleral cartilage cysts in lumpfish, only CD10 antibodies demonstrated true positive staining, supported by genetic and bioinformatic results confirming a lumpfish CD10 orthologue. Moreover, CD10 immunoreactivity was detected in normal noninfected lumpfish head kidney and retinal tissue as well as in tissue from animals infected with *V. anguillarum*, a pathogen completely different from myxozoans. These results strongly support that the anti-CD10 monoclonal antibody is indeed staining a lumpfish CD10 orthologue epitope.

Here, we provide evidence supporting the presence of a host immune response in association with subclinical myxozoan infection of scleral cartilage of otherwise healthy wild lumpfish of different ages sourced from different geographical locations of the Northwest Atlantic. Whole mount staining confirmed that the scleral skeletal cysts, which are observable at a gross anatomical level as multiple discrete pockets of densely packed soft cellular like material in the scleral skeleton (**Figures 2A–G**) are embedded in cartilage rather than bone since these areas do not stain with the Alizarin red bone stain (**Figures 2H, I**). Neither wild or cultured lumpfish scleral cups showed any appreciable histological staining with Alizarin red for bone, indicating that lumpfish in general likely lack scleral ossicles. Franz-Odendaal has proposed a relationship between the configuration of the scleral skeleton and swimming depth of teleosts in which deep sea animals are more likely to lack scleral ossicles (21, 30, 55). Previous studies showing that lumpfish are known to frequent deeper water (14–18) as well as our results herein showing no evidence of scleral ossicle development in lumpfish are consistent with the proposals of Franz-Odendaal.

Our results provide four lines of evidence that wild lumpfish *M. albi* cysts contain host immune components. First, our results using Giemsa and basic fuchsin / toluidine blue differential histochemical staining (**Figures 3****–****5**) suggest the presence of leukocytic cells within the scleral cartilage cysts of wild lumpfish where we detected *M. albi* (**Figure 6**). The cells we observed in the cyst tissue resembled the leukocytes described in previously published histochemical studies by Boomker et al., and Mumford et al. (56, 57). Second, we detected the *M. albi* 18S ribosomal RNA [as previously reported by Cavin *et al*., 2012 (25)] in scleral cartilage cup tissues collected from wild but not in cultured lumpfish, the latter of which do not harbor scleral cartilage cysts. This result supports the notion that an immune response to pathogen invasion, presumably involving host immune cells such as leukocytes, is occurring in the *M. albi* cysts. Third, CD10, which is known to be a marker for hematopoietic progenitor cells in mammalian bone marrow (51), is present in lumpfish (**Figures 8****–****11**) and is expressed in small round cells in the scleral cartilage cysts of wild lumpfish (**Figure 12**). Forth, specific staining for IgM was found in both cell-focused and more diffuse staining patterns in the scleral cartilage cysts (**Figure 7**). The IgM staining was specific since pre-absorption of the anti-lumpfish IgM antibody with purified lumpfish IgM abrogated the staining. Furthermore, western blot of purified lumpfish IgM using this antibody (**Figure S1**) revealed the expected heavy and light chains and the various dimers formed by lumpfish IgM, further confirming the specificity of the anti-lumpfish IgM antibody. These results are consistent with those obtained using an anti-lumpfish IgM antibody described by Bilal et al. (58).

Future studies will be needed to explore and define if the CD10+ cells in lumpfish scleral cartilage cysts are indeed hematopoietic or express other leukocyte characteristics. However, taken together, our collective evidence supports the hypothesis that scleral cartilage cysts commonly develop within the scleral cartilage of wild lumpfish and are composed of a mixture of different host cells of leukocytic lineages and spores or other life cycle material related to a subclinical myxozoan infection. The high number of CD10+ cells in myxozoan infected ocular cartilage tissue raises questions about the level of specificity of this immune response. While lumpfish are known to express genes coding for components of specific immune responses (i.e., as mediated by T and B lymphocytes) and have been demonstrated to mount measurable specific immune responses (42, 47, 59, 60), less is known about innate immune responses in lumpfish. However, lumpfish B lymphocytes are known to be phagocytic (60), suggesting that unique forms of innate immunity could exist in lumpfish. It remains possible that the large CD10+ cell presence in *M. albi* cysts represents a component of a nonspecific innate immune response. Future studies will be needed to explore this.

The absence of scleral cartilage cysts with leukocytic-like tissue and CD10 expression in lumpfish cultured under aseptic conditions at JBARB suggests that CD10 might be useful as a biomarker for myxozoan infection in lumpfish. Since none of the wild animals we analyzed in this study had exophthalmos or other overtly detectable clinical grade disease, our results suggest that lumpfish might uniquely develop an intra-ocular host response to stabilize or contain myxozoan damage to scleral cartilage and other eye structures.

As mentioned previously, *V. anguillarum* infects the eyes of lumpfish and causes exophthalmia. Therefore, we also evaluated the presence of CD10+ cells and IgM in the ocular tissues of lumpfish vaccinated and non-vaccinated after challenge and infection, respectively. While scleral cartilage, *rete mirabile* vascular elements and retinal layers appear relatively undisturbed in vaccinated and *V. anguillarum* challenged animals (**Figures 13A, C**), non-vaccinated infected animals show non-artifactual loss of normal retinal layers, thickening and disorganization of retinal tissues (**Figures 13B, D**) and large numbers of CD10^+^ cells associated with these retinal lesions (**Figures 13D, G**), similar to those we described previously as various forms of retinopathy (61–63). Moreover, areas of putative chondrogenesis or dystrophic mineralization observed in one of the non-vaccinated infected animals (**Figure 13E**) closely coincide with areas of CD10 expression (**Figure 13F**) reminiscent of the leukocytic infiltration comprised of mast cells, macrophages, T and B lymphocytes and neutrophils during auricular chondritis in cat (*Felis catus*) cartilage regeneration as described by Wilson and colleagues (22). An increased level of IgM expression was present in the retinal lesions in the non-vaccinated infected animals compared to the vaccinated challenged animals (**Figures 13J, I**, respectively)). Our results suggest that the immune interaction with the pathogen, either bacterial or myxozoan, increases CD10+ cells, IgM+ cells and IgM in the ocular tissue. Our results support the idea that normal lumpfish ocular CD10 expression changes in response to pathogens and that CD10+ cells are recruited to regions of intraocular disease in an ocular immune response to pathogens.

The expression of CD10 and its co-expression with other markers such as ASMA in lumpfish tissues prompts additional questions. CD10 marks hematopoietic progenitor cells in mammalian bone marrow (51) and is considered to play a functional role in development of stem cells (44). Other studies have shown that CD10 expression by stromal cells is involved in maturation niches mediating B lymphocyte development in bone marrow (64). There is also evidence that CD10 plays a role in chondrogenesis (65, 66). Herein we observed specific CD10 expression in numerous small cells in follicular regions of the head kidney (**Figure 11A**) and in basal cells of the renal tubules (67) of the head kidney (**Figure 11B**). Zebrafish renal tubule tissue contains stem cells that can act to regenerate kidney throughout life (68) but we do not know if these cells express CD10 or if the CD10^+^ cells that we observed in lumpfish renal tubules are stem cells. In normal adult lumpfish eye, CD10 is expressed in a well-defined peri-photoreceptor pattern in the outer layers of the retina and in well-defined bands in the inner layers of the retina which, upon double IHC, is observed to largely colocalize with ASMA (**Figures 11C–F**). The colocalization of CD10 and ASMA in normal lumpfish retina is intriguing but requires further study. The CD10 expression in normal retina is also reminiscent of the expression of CD10 in sable fish brain (47). Since ASMA is known to be expressed by neural crest cell derived lineages (69, 70), further work could explore if ASMA-like expression in *C. lumpus* retina is functionally related to CD10 or other properties of retinal nervous tissue in health and disease states. The size and shape of the CD10^+^ cells in the scleral cartilage cysts best matches the phenotype of “possible precursor cells” but could also represent lymphocytes, macrophages or granulocytes previously described in lumpfish (42). Another CD10 expression pattern that we observed in the lumpfish eye was in cells of a more stromal or dendritic morphology (64). Beyond its well-known classical role in hematopoiesis, the dysregulated expression of CD10 (otherwise known as neprilysin, membrane metallo-endopeptidase, neutral endopeptidase, common acute lymphoblastic leukemia antigen), is known to be associated not only with leukemia but also with ocular and neurological diseases in mammal (44, 71–73). Much remains to be discovered about the characteristics and roles of CD10 in both normal and pathogen-bearing lumpfish tissues.

To conclude, we propose that lumpfish possess a novel IgM and CD10^+^ cell-related intraocular response to pathogens in order to mitigate damage to eye structures. Recent studies have demonstrated that IgT-B cells play a key role in fish mucosal immune responses to parasitic and bacterial pathogens (63). Future studies could assess if IgT-B cells contribute to a putative ocular specific immune response to infection by pathogens. A recent national level analysis of cleaner fish use on salmon farms in Norway concluded that, while benefits of the use of cleaner fish on some farms is clear, suboptimal usage of cleaner fish remains in some settings (74). Our results raise the possibility that pathogen mediated visual disruption might impact cleaner fish visual health in such settings. While our results provide some examples whereby subclinical opportunistic disease might be manageable by wild lumpfish, they also raise the more broadly important questions of how subclinical disease processes impact normal visual function and survival of lumpfish in the wild or efficacy as pest biocontrol.

## Data Availability Statement

The datasets presented in this study can be found in online repositories. The names of the repository/repositories and accession number(s) can be found in the article/**Supplementary Material**.

## Ethics Statement

The animal study was reviewed and approved by Institutional Animal Care Committee Memorial University.

## Author Contributions

Conceptualization: RG, JS, HP, RA, WG, KK, SK, IV. Methodology: RG, JS, HP, RA, SK, IV, TC, AH, SC, KV, DB. Investigation: RG, HP, RA, IV, TC, KK. Writing—original draft: RG, JS, HP, KK, WG, SK. Resources: RG, HP, JS, DB, KK, DB. Writing—Review and Editing: RG, JS, HP, KK, WG, SK, IV, TC, AH, SC, KV. Visualization: JS, RG. Supervision: RG, HP, JS. Funding acquisition: RG, JS, HP. All authors contributed to the article and approved the submitted version.

## Funding

This work was funded through grants from the Vitamin Research Fund, Ocean Frontier Institute (2017), Canada First – Ocean Frontier Institute (Module J3), Medical Research Foundation of the Faculty of Medicine (2016), through leverage funds from School of Graduate Studies, Memorial University, NSERC-Discovery (RGPIN-2018-05942), and through support from the Dr. Joe Brown Aquatic Research Building (JBARB). Canada Foundation for Innovation.

## Conflict of Interest

The authors declare that the research was conducted in the absence of any commercial or financial relationships that could be construed as a potential conflict of interest.
