# Supplementary material for: CD10+ Cells and IgM in Pathogen Response in Lumpfish (Cyclopterus lumpus) Eye Tissues
Source: Front Immunol. 2020 Nov 20;11:576897. doi: 10.3389/fimmu.2020.576897 (PMC7714965; doi:10.3389/fimmu.2020.576897)
Supplement: Supplementary file 1 [file DataSheet_1.pdf]

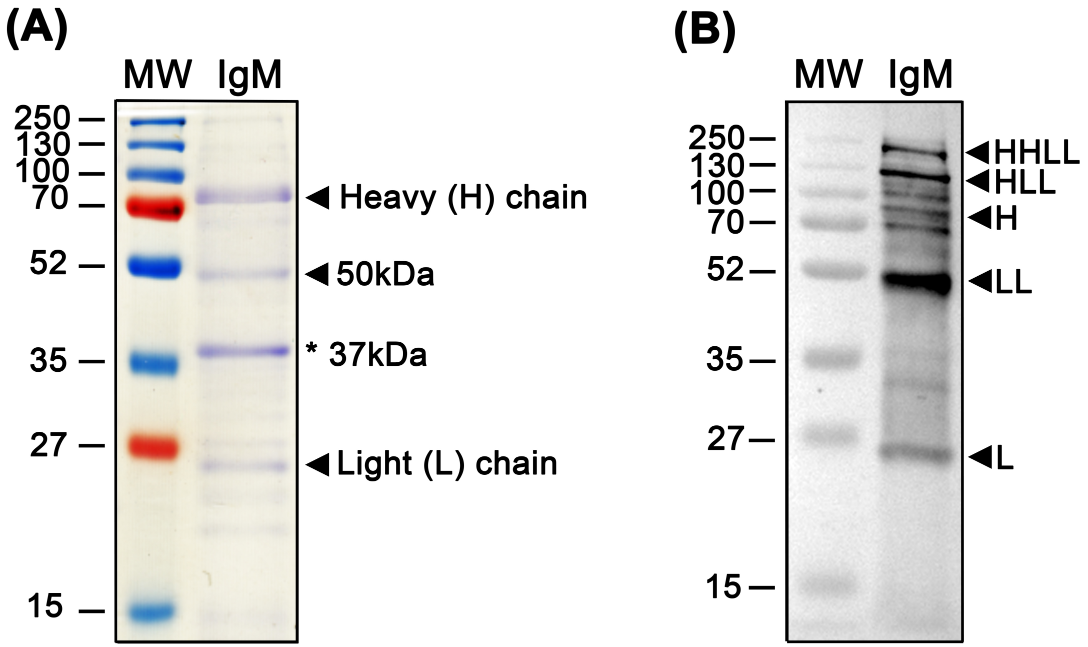

Figure S1. (A) Coomassie blue staining of purified lumpfish IgM (IgM) analyzed by 12% sodium dodecyl sulfate - polyacrylamide gel electrophoresis (SDS-PAGE) under reducing conditions relatively to pre-stained molecular weight (MW) markers of relative molecular weight indicated on the left in kDa. Major bands observed in purified IgM were 75kDa (Heavy chain), 50kDa, 37kDa and 25kDa (Light chain). * non-specific band. (B) Western blot of purified lumpfish IgM (IgM) using chicken IgY anti-lumpfish IgM primary antibody and goat anti-IgY-HRP. The major bands observed in purified IgM by Coomassie blue staining were also detected with the anti-IgM antibody including the 75kDa (heavy chain: H), 50kDa (possible light chain homodimer: LL), and 25kDa (light chain: L) but not the 37kDa band. Additional higher molecular weight bands of approximately 100kDa, 125kDa and 204kDa were also observed by western blot and could represent various complexes of the IgM chains: HL, HLL and HHLL, respectively.
